# Evaluating a New Short Self-Management Tool in Heart Failure Against the Traditional Flinders Program

**DOI:** 10.3390/jcm13226994

**Published:** 2024-11-20

**Authors:** Pupalan Iyngkaran, David Smith, Craig McLachlan, Malcolm Battersby, Maximilian de Courten, Fahad Hanna

**Affiliations:** 1 Melbourne Clinical School, University of Notre Dame, Melbourne, VIC 3000, Australia; pupalan.iyngkaran@students.torrens.edu.au; 2 Centre for Healthy Futures, Torrens University Australia, Surry Hills, NSW 2000, Australia; craig.mclachlan@torrens.edu.au; 3 College of Medicine & Public Health, Flinders University, Adelaide, SA 5042, Australia; david.smith@flinders.edu.au; 4 College of Medicine and Public Health, Flinders University, Norwood, SA 5067, Australia; malcolm.battersby@flinders.edu.au; 5 Australian Health Policy Collaboration, Institute for Health and Sport (IHES), Victoria University, Melbourne, VIC 8001, Australia; maximilian.decourten@vu.edu.au

**Keywords:** clinical treatment, heart failure, Flinders Program, risk assessment, self-management, readmission

## Abstract

**Highlights:**

**What are the main findings?**

- Chronic disease self-management (CDSM) programs have proven benefits in the management of many chronic diseases, and this study supports new impetus in CHF;
- CDSM’s benefits in improving MACE in CHF remain unclear;
- CDSM can be delivered as generic or disease-specific programs; the former has been tested widely in CHF, this study suggests possibilities for generic programs

**What are the implications of the main findings?**

- Generic CDSM programs can be used in CHF;
- Generic short-form tools derived from gold-standard CDSM can risk-stratify poor and good self-managers.
- Self-managers with borderline and average abilities require greater understanding when designing randomized trials to further analyze these findings.

**Abstract:**

**Background/Objective:** Heart failure (HF) is a complex syndrome, with multiple causes. Numerous pathophysiological pathways are activated. Comprehensive and guideline-derived care is complex. A multidisciplinary approach is required. The current guidelines report little evidence for chronic disease self-management (CDSM) programs for reducing readmission and major adverse cardiovascular events (MACE). CDSM programs can be complex and are not user-friendly in clinical settings, particularly for vulnerable patients. The aim of this study was to investigate whether a simplified one-page CDSM tool, the *SCR*eening *in H*eart *F*ailure (*SCRinHF*), is comparable to a comprehensive Flinders Program of Chronic Disease Management, specifically in triaging self-management capabilities and in predicting readmission and MACE. **Methods:** *SELFMAN-HF* is a prospective, observational study based on community cardiology. Eligible patients, consecutively recruited, had HF with left ventricular ejection fraction <40% and were placed on sodium–glucose co-transporter-2 inhibitors (SGLT2-i) within 3 months of recruitment. SGLT2-i is the newest of the four HF treatment pillars; self-management skills are assessed at this juncture. CDSM was assessed and scored independently via the long-form (LF) and short-form (***SF***) tools, and concordance between forms was estimated. The primary endpoint is the 80% concordance across the two CDSM scales for predicting hospital readmission and MACE. **Results:** Of the 117 patients, aged 66.8 years (±SD 13.5), 88 (75%) were male. The direct comparisons for ***SF*** versus ***LF*** patient scores are as follows: “good self-managers”, 13 vs. 30 patients (11.1% vs. 25.6%); “average”, 46 vs. 21 patients (39.3% vs. 17.9%), “borderline”, 20 vs. 31 patients (17.1% vs. 26.5%), and “poor self-managers” (vulnerable), 38 vs. 35 patients (32.5% vs. 29.9%). These findings underscore the possibility of *SF* tools in picking up patients whose scores infer poor self-management capabilities. This concordance of the ***SF*** with the ***LF*** scores for patients who have poor self-management capabilities (38 vs. 35 patients *p* = 0.01), alongside readmission (31/38 vs. 31/35 *p* = 0.01) or readmission risk for poor self-managers versus good self-managers (31/38 vs. 5/13 *p* = 0.01), validates the simplification of the CDSM tools for the vulnerable population with HF. Similarly, when concurrent and predictive validity was tested on 52 patients, the results were 39 (75%) for poor self-managers and 14 (27%) for good self-managers in both groups, who demonstrated significant correlations between ***SF*** and ***LF*** scores. **Conclusions:** Simplifying self-management scoring with an ***SF*** tool to improve clinical translation is justifiable, particularly for vulnerable populations. Poor self-management capabilities and readmission risk for poor self-managers can be significantly predicted, and trends for good self-managers are observed. However, correlations of ***SF*** to ***LF*** scores across an HF cohort for self-management abilities and MACE are more complex. Translation to patients of all skill levels requires further research.

## Highlight

## 1. Introduction

Heart failure (HF) is a complex cardiovascular condition, arising from various contributing factors and typically accompanied by multiple comorbidities [1,2]. This multifaceted nature makes its management challenging for both healthcare providers and patients. Delivering comprehensive and optimal HF management requires several factors to be considered: first, the complex pathophysiology, with an attempt to improve function with medical treatments; second, a systems approach utilizing a multidisciplinary team to administer an integrative guideline-based management program (GDMT). The goal is maintaining quality of life, limiting preventable deteriorations (morbidity and mortality), and managing health resources [3,4]. There remain population-level gaps in preventing major adverse cardiovascular events (MACE), including hospital readmissions [5,6,7,8,9].

Chronic disease self-management (CDSM) is a health services approach and a domain within HF programs. In theory, CDSM can be a cost-effective approach to bridging gaps in HF care. While CDSM studies for most chronic diseases have reported positive outcomes [10,11], studies in HF have been relatively disappointing. In fact, recent guidelines have demoted this therapy in terms of both performance and quality measures, citing a lack of evidence [9]. CDSM tools can be complex. Their clinical roles can be difficult to navigate in clinical settings. There are structural issues with CDSM with respect to HF management. These include a lack of randomized trial evidence demonstrating efficacy [9] and a lack of clinical understanding on the value of CDSM [9,12] and how it fits within disease management domains [13], raising the question: could an important gap in CDSM programs for HF be within the tools themselves? The **F**linders **P**rogram of Chronic Condition Management [*Flinders **P**rogram (***FP^®^****)*] is a gold-standard generic tool [14]. This generic unmodified tool, in its long form (***LF***), is the foundation from which this study is based. Short forms (***SF***) are uncommon and have not been tested in an HF population. Our team has demonstrated via a press publication the psychometrics of ***FP*** in ***CHF*** syndromes.

The **SELF**-**MAN**agement in **H**eart **F**ailure ***(SELFMAN-HF)*** study [14] was derived to address one critical issue: can a novel ***SF*** CDSM tool (*SCRinHF) be validated against the established and best-available ***LF*** in determining readmissions and MACE? To date, there are no proven CDSM tools that have been adapted from its generic form to a shorter version, and this includes HF. Numerous studies have concluded that the theoretical basis for CDSM is itself well established; the methods of delivery have been well studied, and these tools are the best means of delivering CDSM programs [10,15]. With a changing healthcare landscape, and with evidence for HF lacking, a novel simplified approach could be explored. There was also impetus from existing examples of simplifying other chronic disease tools—e.g., SF-36 to SF-12 [16] and PACIC to PACIC-plus [17]—for initiatives in HF [18,19,20].

### Aims

Our aim was to validate the ***SF*** [14] against the established ***LF*** with respect to self-management capabilities, a primary endpoint of hospital readmission and a secondary outcome of MACE. 

## 2. Materials and Methods

Detailed references for this study protocol, design, and methodology have been published [14]. The ***SELFMAN-HF*** is a prospective, observational case–cohort study that examined the feasibility and validity of the ***SF*** tool, comparing the novel one-page tool to the ***LF*** [14]. In brief, study patients were consecutively enrolled as they presented as community outpatients. Validity was explored with comparisons to the ***LF*** in a range of measures including self-management, clinical progress, and predicting 12 months MACE, including hospital readmissions. Feasibility was not a planned aim in this study.

### 2.1. Participants

The community service has 6 clinical sites treating HF. Participants were recruited and enrolled in one clinical site in Western Melbourne. A minimum of 80 patients with HF who met the inclusion criteria were offered the opportunity to participate. Eligible patients aged over 18 commenced sodium glucose cotransporter-2 inhibitor (SGLT-2I) within 3 months for systolic HF [echocardiographic ejection fraction (EF) < 40%]; they received care within the predefined health jurisdiction. Patients were excluded if concerns were raised by any medical staff; if they had a life expectancy of ≤6 months; or if they were receiving palliative or nursing home care. Patients of variant cognitive statuses and patients with dementia were not excluded if consent from a caring relative or legal guardian who would assist in the completion of the study was given. This study also excluded individuals with significant neurological or cognitive impairments, those unable to provide written informed consent for any reason, clients who did not typically reside in the region (preventing follow-up data collection), and patients for whom the dates of SGLT-2I prescriptions were unknown.

* ***SCR***eening ***in H***eart ***F***ailure *(**SCRinHF)** is designed as a* simplified triage tool *to risk-stratify CDSM capabilities and complement gold-standard tools.* It is compared against the **F**linders **P**rogram of Chronic Condition Management [*Flinders **P**rogram (**FP^®^**)], a gold-standard generic tool. **SF** (short-form tools) and **LF** (long-form tools) will be the acronyms used to describe the tools and programs in this paper.

### 2.2. Sample Size Calculation

See Section 2.6.

### 2.3. Trial Instruments, Procedures and Treatment

#### Short-Form (SF) and Long-Form (LF) Tools (Flinders Program or FP^®^) Questionnaire

The **F**linders **P**rogram (***FP^®^***) is a generic and comprehensive CDSM program that utilizes 4 tools to obtain a patient-centred and codesigned comprehensive and flexible program of care for patients [21]. The ***FP^®^*** is a gold-standard generic chronic disease management program and is on par with other CDSM programs in achieving outcomes; i.e., it assess self-management understanding and goals, and from this, an education and care plan can be tailored to achieve self-efficacy in managing chronic disease [21,22]. The tools used by the ***FP*** extract patient-reported outcomes (PRO) and health service data along 4 domains. A 2-day course certifies health workers to use the ***LF*** to conduct interviews with clients, which can take up to 90 min. The ***LF*** tools include the Partners in Health Scale (PIH), a self-rated questionnaire for the patient to assess 4 domains, namely, self-management knowledge, attitudes, behaviors, and the impacts of their chronic condition (Table 1); and the Cue and Response Interview (C&R), a health-worker-administered tool that explores the same PIH questions via open-ended questions and responses, rated from the health provider’s perspective. The final 2 domains are not relevant for this study [21]. The programs questionnaire estimate a baseline and utilize other planning tools to attain the desired CDSM goal. 

The ***SF*** tool is based on the principles of CDSM programs [14,21,22,23,24] and aims to simplify its use and broaden the usability of the application across the health continuum. The ***SF*** tool aims to assist with triaging patients at any health encounter along several domains, and self-management capabilities are one key domain (Table 1). This is unique and important as CDSM discussions that are often relegated to an exclusive aspect of a patient’s health journey can now begin during any health encounter. Studies have shown that the ***LF*** can assess and deliver one client’s capacity to self-manage, while the program then provides a generic set of tools in a structured process that enables health workers and patients to develop health goals collaboratively [14,21]. The ***SF*** tool is scored by the enrolling trial nurse based on information already recorded on the patient, with clarifications as needed. It does not require a lengthy patient interview [14].

**Table 1:** The CPFI, Partners in Health scale, and SCRinHF details and scoring.

A. PIH Domain	PIH Scale	SCRinHF Domain	SCRinHF Question
**a.** **Knowledge**	1. Overall, what I know about my health condition(s). 2. Overall, what I know about my treatment, including medications for my health condition(s).	1. Self-care maintenance/monitoring	a. Do you know “how to (skill)…to achieve (goal)”… 1. Problem Solve—e.g., (i) monitoring; 2. Decision making question 3. About Physical function—e.g., (i) exercise
**b.** **Partnership in treatment**	3. I take medications or carry out the treatments asked by my doctor or health worker. 4. I share in decisions made about my health condition(s) with my doctor or health worker. 5. I am able to deal with health professionals to get the services I need that fit with my culture, values and beliefs. 6. I attend appointments as asked by my doctor or health worker.	2. Self-management	b. Do you know “what to do if (skill) to achieve (goal)”… 4. Resource utilization e.g., (i) monitoring with action 5. Form patient provider partnership e.g., (i) engage health system 6. Action planning when self-tailoring
**c.** **Recognizing and managing symptom**	7. I keep track of my symptoms and early warning signs (e.g., blood sugar levels, peak flow, weight, shortness of breath, pain, sleep problems and mood). 8. I take action when my early warning signs and symptoms get worse.	3. Self-care efficacy/confidence	c. Do you know “how confident you are (skill)…when faced with (goal)” 7. Has client previously received Rehab/education? State level of Self-Care Confidence (SR, SE, TI, TE)—e.g., (i) adherence to diet ii) compliance
**d.** **Coping**	9. I manage the effect of my health condition(s) on my physical activity (e.g., walking and household tasks). 10. I manage the effect of my health condition(s) on how I feel (i.e., my emotions and spiritual well-being). 11. I manage the effect of my health condition(s) on my social life (i.e., how I mix with other people). 12. Overall, I manage to live a healthy life (e.g., no smoking, moderate alcohol, healthy food, regular physical activity and manage stress).	Operator assessment of care	Covered in another section of tool.
**B. Correlation of PIH and SCRinHF—the column below highlights our approach to matching the domains and scores.** ***@At this point no correlation exists or has been tested between CFPI and SCRinHF*. This process will also require validation.**			
**Scoring**	Likert 0–8 Total 96	SCRinHF domain 1 2 3	PIH correlation a, d (6 questions) b, c (6 questions) a, b, c, d (12 questions)
**Interpretation**	SCRinHF is a risk score. Scores 0 indicated low risk, i.e., at least average ability to perform self-management task. Self-efficacy is more rigorous and compares to the entire PIH score. The SCRinHF is compared to health-staff-administered PIH scale or C&R.	***@SCrinHF Score*** 0 Good 1 Average 2 Borderline 3 Poor	***@PIH Pass Score*** >8/16 (domain a or c—2 questions/domain) >16/32 (domain b or d 4 questions/domain) >48/96 (combined CFPIscore)
**Validation**	Construct and factor validity. Face and content validity. Criterion related validity (concurrent, predictive).	Ref [25] Delphi process	Research design and conduct. Expert, patient consultation with study data. Association to gold standard, predict outcomes.

(A) The Partners in Health scale (PIH) is a patient-reported outcome tool and the first part of The Flinders Program. Four domains are assessed. In contrast, the SCRinHF extracts three components; the coping domain is rested here and extracted in another tool domain. (B) The PIH is scored 0 to 8 in each care dimension and, overall, achieves a score of 96. With respect to scoring, the SCRinHF domain 1 and 2 correlate with the respective PIH domains. The third domain of self-effectiveness correlates with all PIH domains. Each SCRinHF domain has a score of 0 (competent) or 1 poor. In interpretation, SCRinHF patients can have a combined score of 0, 1, 2, or 3. A score of 3 equates to a PIH total score of <48. Individually, a score of 1 in each SCRinHF domain will equate to <16 for the PIH domains. Pass scores in PIH are >4/8 per question, >8/16 or 16/32 for specific domains, or >48/96 overall are not being assessed.

### 2.4. Data Collection

#### 2.4.1. Comparing the Scores of Self-Management Domains

***SF*** has 4 steps [14] and works on the basis of the following (Table 1): first (domain 1), understanding a client’s generic baseline readmission risk; second (domain 2), determining the buffers to counter this risk; this includes three parts, patients’ living-at-home skills and goals determined via 3 questions on self-management maintenance, 3 on self-management, and 1 on self-management confidence or efficacy; third (domain 3), support for living at home; fourth, (domain 4) the chronology is employed as an introspective element when understanding where a client is and where they should aspire to be in the context of their journeys. The scoring system (domains 5 and 6) has not been tested clinically. The rationale for scores are matched to ***LF*** scales and are described in [14]. 

#### 2.4.2. Scoring of Outcomes

MACE was measured at 6 and 12 months as the number of planned and unplanned hospitalizations, cardiovascular events, and mortality. Side effects are listed as those in the manufacturers’ brochure and documented as reducing the dose, ceasing medications, and worsening renal function [WRF: 25% reduction in eGFR or increase in serum Creatinine (SCr)]. Attendance was calculated, in years, from the day of starting the SGLT-2 inhibitor and documented as visits for test and clinical review as per the number of clinical bookings and non-attendance. 

#### 2.4.3. Scoring of Established Tools (Appendix)

Baseline scores were completed and presented as per published tool guidelines. Multiple tools measuring well-being were used. The references for its clinical justification are provided as follows: PIH [14,21,22,23,24,26,27,28,29,30,31], Charlson Comorbidity Index (CCI https://www.mdcalc.com/calc/3917/charlson-comorbidity-index-cci, accessed on 17 May 2024) [27], SF-12 (https://orthotoolkit.com/sf-12, accessed on 17 May 2024) [28], and PHQ-9 [29]. The scoring is described in the results section (Table 1).

#### 2.4.4. Scoring of the ***SF*** and the ***LF***

The ***SF*** tool extracts 3 CDSM domains from the PIH scale and allows for health staff to score in a binary fashion. Specifics are provided in Table 1, published methods [14]. Client (or dyad) contribution in PRO tools makes up a component of the non-controlled information extracted from carers and health systems. Thus, with any scoring, a degree of judgement is required, and the removal of the 8-point Likert scores challenges health staff to commit to clinical decision making

### 2.5. Ethical Considerations

This study has been approved by the St Vincent’s ethics committees committee (approval no. LRR 177/21). All participants completed a written informed consent form prior to enrolment in the study. The study results will be disseminated widely via local and international health conferences and peer-reviewed publications.

### 2.6. Statistical Aspects and Data Analysis

The study cohort assesses validity, which will allow for the appropriate parameters to determine sample size power calculations for future studies that will utilize a controlled design. Initial data analysis will investigate the distribution characteristics of each primary and secondary outcome measure and determine either a parametric or semi-parametric statistical approach to the main data analysis. Descriptive statistics for baseline demographics and clinical characteristics will be presented as means (standard deviation) for continuous data and count (%) for categorical data. As an example, the previous publication provides estimates for pilot sample size calculations for categorical data [32,33,34,35,36]. Latest-edition IBM SPSS (2024) Statistics 29 has been utilized for this analysis.

## 3. Results

**Figure 1:**
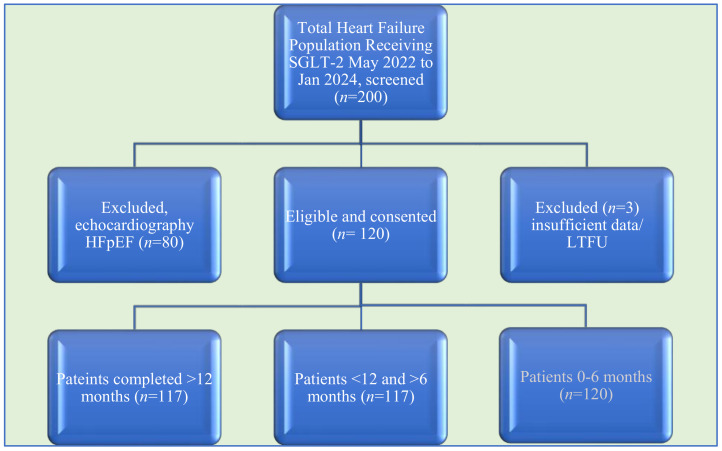
Study flowchart. Abbreviations: f; HFpEF—heart failure with preserved ejection fraction; LTFU—lost to follow-up; n—number of patients.

From May 2022 to January 2024, 210 patients were screened for SGLT-2I, with 120 patients heart failure with reduced ejection fraction (HFrEF) consenting to participate in this study (Figure 1). All patients completed baseline criteria and were enrolled. At the time of final follow-up and study closure, no patient withdrew consent; however, nine patients were lost to follow-up. Their reasons for being lost to follow-up were confirmed by a relative or their general practitioner, including travel overseas (*n* = 2), relocating (*n* = 2), mental health (*n* = 1), and no contact being made (*n* = 4).

### 3.1. Baseline Characteristics

The study population comprises 117 patients. Baseline study characteristics are summarized in Table 2 and Table 3. The population was 66.8 years old (SD: 13.5); 88 (75%) were male and 29 were female. The majority of patients ethnicity were Caucasian [90 (77%)], followed by South Asian, Asian, African, or Indigenous. Most patients were married [75 (64%)]. At least 71 (65%) described their spouses as family support. A majority of 90 (76.9%) patients had received an education up to high school level. As many as 30 (25.6%) of patients did not record any associated comorbidity at baseline, 26 (22.3%) had one, and the remainder had three or more. Hypertension and hypercholesterolemia were the most common comorbidity, being present in 79 (68%) and 73 (62.4%) patients, respectively. Renal impairment, defined as eGFR < 60 mL/min, was recorded in 48 (39%) patients. Coronary artery disease (CAD) and diabetes were recorded in 51 (44%) and 42 (36%) patients, respectively. Obstructive sleep apnea was also common, recorded in 31 (26.5%) patients in the cohort. Smoking history was recorded in 53 patients (45%). CHF was not a new diagnosis, i.e., a chronic condition, having been diagnosed prior to 12 months in 34 (29%) of the cohort. Further details are described in Appendix A.

### 3.2. Self-Management Scores in the Cohort

The ***LF*** scores are highlighted in Table 4. For the 117 patients, the number and scores for knowledge (K), coping (C), partnership in treatment (P), and management and recognition of symptoms (M) are scored as 16, 32, 32, and 16 points, respectively, for a maximum of 96 points. With the ***SF*** domain 1, for self-maintenance (*Ma)* 76 patients (65%) scored as good, and 41 patients (35%) scored as average to poor. In comparison, the ***LF*** scores combining K and C at similar levels are 15 (12.8%) for good and 27 (23.1%), 48 (41.9%), and 27 (22.2%) average to poor. For ***SF*** dimension 2, or self-tailoring (*M*x), 61 (52.1%) showed good capabilities, while 56 (48.9%) of patients were average to poor. In comparison, the ***LF*** scores combining P and M at similar levels are 35 (29.9%) for good and 43 (36.8%), 26 (22.2%), and 13 (11.1%) for average to poor. When both tools are looked at in combination, the ***SF*** has the capacity for a wider scoring range. The direct comparisons for ***SF*** versus ***LF*** are as follows: good, 13 vs. 30 (11.1% vs. 25.6%); and average, 46 vs. 21 (39.3% vs. 17.9%), 20 vs. 31 (17.1% vs. 26.5%), and 38 vs. 35 (32.5% vs. 29.9% [*p* < 0.01]*). 

### 3.3. Readmission and MACE

**Figure 2:**
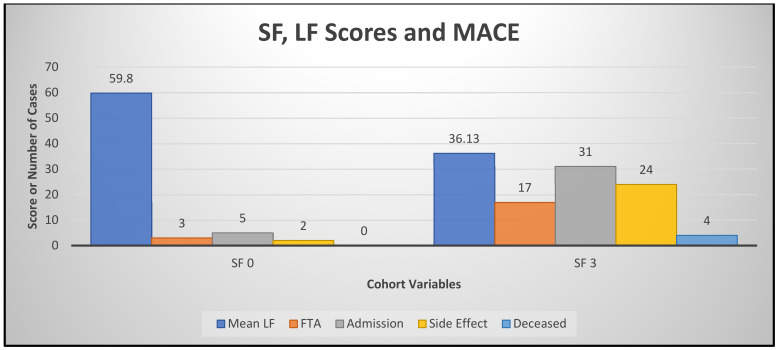
SF and LF correlation with readmission and MACE.

Among 117 patients, 6 patients died within the 12-month follow-up. One patient had cardiac amyloid and renal failure, two from complications of CHF or cardiac procedure (who were 86 and 92 years old, respectively), and three had CHF secondary to illicit drug use. The total number with five or more hospital admissions came to 14 (12.0%) over the 12-month period. Among these, 2 patients had terminal cancer, several were elderly with dementia and other requiring cardiac and non-cardiac care; 31 (26.5%) had between one and three admissions; 17 (14.5%) had one admission; and 55 (47%) did not have an unplanned cardiac or non-cardiac admission. In terms of attendance to the clinic, 34% failed to attend >25% of pre-booked appointments. These patients had the highest readmission rates. Reasons documented for cardiovascular readmissions include HF (17), cardiovascular reasons (21), AF (9) (AF ablation and cardioversions), and 13 had angiography, device insertion, and bypass surgery. Adverse events, including reduced, ceased, and worsening renal function, as well as other medication side effects, occurred in 53 (45.2%) of the patients. Among the 38 patients who scored 0 (*n* = 13) or 3 (*n* = 38) in *SF*, 4 vs. 0 died, and 4 vs. 1 were lost to follow-up; unplanned cardiovascular admissions, including HF and death, amounted to 5 vs. 31 (*p* < 0.01). Repeat admissions of >5 were seen in 5 vs. 1 patients, and between 1 and 3 readmissions were seen in 13 and 2 patients, respectively. The breakdown for admissions for poor and good self-management scores is highlighted in Table 5 and Figure 2. 

### 3.4. Validity

Concurrent and predictive validity was tested on 52 patients’ data (Appendix A). These patients comprised 39 poor and 14 good self-managers, both of which demonstrated significant correlations between ***LF*** (PIH scale) and ***SF*** scores. For concurrent validity, an association was noted between ***LF*** and ***SF*** when adjusting for age, sex, and the number of co-morbidities. Multivariate analysis shows that the ***LF*** (PIH scale) score correlates with ***SF*** [with coefficient −14.692, SE 4.268217; t = −3.44; *p* > 0.001 (95% CI −23.27855 to −6.105458)]. For predictive validity from 48 observations, similar associations were not demonstrated [coefficient −1.327082, SE 1.039514; t = −1.28; *p* > 0.209 (95% CE −3.423462 to 0.7692982).

## 4. Discussion

This study has shown that it is feasible to simplify established CDSM principles as a triage tool, and they can predict readmission and probably MACE. The ***SF*** tool, a binary questionnaire with a pooled score ranging from 0 to 3, correlates significantly with the ***LF*** in identifying poor self-managers (a vulnerable patient cohort), who are characteristically at a significant risk of readmissions. This tool displayed some trends with respect to good self-managers in predicting lower readmission and MACE, albeit with gaps as assessed with the ***LF***. Patients who were borderline or average self-managers did not show similar correlations.

Concerning the study’s stated aim of predicting self-management capabilities based on ***SF*** and ***LF*** scores, our study found that patients who score poorly (poor self-managers) in most domains of the ***LF*** correlate with a matched “poor” score in the ***SF***. What appears more ambiguous are the borderline and average cases, where patients have a range of abilities. The ***SF*** tool missed 3/5 good self-managers, which raises the issue of the sensitivity of binary scoring in extrapolating broader nuances in CDSM capabilities. This raises the possibility of a role for confounders here, which could include patient factors, supports, previous CDSM education, and a range of other factors. An area that is receiving greater attention is the role of dyads [37]. If we look at a previously published experience on short forms, interestingly, in an older publication of SF-36 scores (noted despite the loss to patients’ response opportunities), the authors found that SF-36 binary recoding provided the possibility of a newer, easier, smarter method to administer, compile, and score tests and process data [38]. In 1248 HF patients who completed a PRO tool [Kansas City Cardiomyopathy Questionnaire-12 (KCCQ-12)], Sandhu et al. found that with respect to the correlation with the four-scoring item NYHA class and KCCQ-12, patients’ perceptions were greatest when clinicians accessed patients’ KCCQ-12 scale [25]. Another study in support of multi-response PRO tools was the FAIR-HF trial of 3459 HF patients with iron deficiency who were randomly assigned to receive intravenous iron (ferric carboxymaltose) or saline (placebo). For the secondary endpoint of HRQoL, using the KCCQ score, scores started at 53 points in both groups. The ferric carboxymaltose group had an improvement of 14 points, and the placebo group of 6 points. This effect was significant in favor of ferric carboxymaltose (*p* < 0.001) [39]. This area will thus require further attention in the ongoing analysis of how and where long- and short-form tools can best be utilized in research and clinical settings. Larger sample sizes and randomization may be required. 

In reference to our other endpoint, readmissions, this study’s findings also showed significant concordance between poor self-managers and higher readmission, and trends correlating good self-managers with lower readmissions. Poor self-managers are a vulnerable group and are naturally associated with a higher risk of readmissions. There are a range of factors, and these includes sociodemographic factors. These can be identified with both the ***LF*** and ***SF*** tools. This finding has significance in today’s HF healthcare resourcing, where readmission is the largest contributor to cost (around 2% of health budgets) [1,3,4]. Furthermore, identifying poor self-managers leads to the identification of behaviors relevant for self-management capabilities, making this an identifiable and modifiable risk factor. The opportunity to optimize GDMT then presents itself, i.e., through triaging these patients at various healthcare encounters and providing these high-risk patients with early, patient-focused direction and longer-term readmission planning. Patient- and resource-centric care seem a feasible objective. Nonetheless, the true value of the clinical tools can only be gauged after they have undergone rigorous validation. The construct validity of the ***PIH*** in HF is established in [40,41]. The next phase is to establish face and content validity via a Delphi process. 

With regards to predictive validity, this study suggests that there are significant observations to support patients who have low scores in self-management skills; and in risk prediction, these are also the patients who are at higher risk of readmission and adverse outcomes. This trend (Appendix A) needs to be tested in a larger sample, and at the next or subsequent stage removal of bias must be undertaken through blinding and randomization. Good and poor self-managers make up 51/117 patients in the cohort. To advance this concept for future consideration, the scoring process is explored. To help interpret the complex scoring process, the range of scores from the ***LF*** in this study ranged from 16 to 79 points. There were differences in domain scores, with the partnership (P) domain being the highest mean score for good (17.67). The knowledge (K) domain appears to be the hardest to score well. Thus, to understand the nuances of ***SF*** scores fully, greater understanding of CDSM domains is needed, as well as of their potential weighted impacts on self-efficacy (i.e., overall self-management abilities). This may well then present an opportunity to understand better the role of simplifying scores and to achieve concordance with ***LF*** across all self-management capabilities. This study, however, was not intended to compare the individual components of each domains score. 

### 4.1. Limitations

This study has several limitations. First, on the issue of the generalizability of the findings, while there are positive signs with respect to observations of the ***SF*** tool’s effectiveness, this study lacks randomization and a control. Any follow-up study will require this design. Second, the types of readmissions include planned and unplanned cardiac or medical procedures, complications, or delays from the planned procedures, as well as other forms of care. This study was not designed to accurately interrogate these confounders in detail. Third, this was an observational longitudinal study; MACE data were presented for the lowest (0, good self-manager) and the highest (3, poor self-manager) groups; i.e., no significant data were observed for the intermediate groups. In future, a larger cohort, multi-site, controlled, and randomized trial would help to factor in the various confounders. Finally, on scoring nurse-aided vs. PRO tools, this tool is only tested on nurse-aided.

### 4.2. Current Findings and Future Research

There are numerous recognized simplifications of established tools. This is the first (that we know of) for the generic CDSM tool. As the concepts and self-management domains are established, it was encouraging to see signals for poor and good self-managers. There are gaps in triaging the intermediate group and also with respect to broader generalizability. Thus, future studies will need to consider the following: First, on self-management capabilities, our observation suggests that the self-management domains, when utilized in the ***S*,** may require weighting; a simple start would be to understand how each of the individual four domains (K, P, M, C) correlates to MACE. Second, in interpreting the association between readmission and MACE, readmission is a complex issue. Uptake of GDMT [3,4] and the hospital care programs processes [5,7] are established, and when implemented, they improve outcome [42,43,44] Heterogeneity (e.g., disease phenotype, comorbidities, certain demographics, and risk factors that play important roles), HF disease chronology and phases, specific identifiable vulnerable phases (including non-HF), and status following HF hospitalizations are established factors [45,46,47,48,49]. In this study, patients were recruited at the point where the diagnosis of HF was already made; however, in 11% of patients, the diagnosis of HF was made more than 5 years before the SGLT-2 was started. At recruitment, many HF pillars (i.e., beta-blockers, RAAS inhibitors, and aldosterone antagonists) were already commenced. However. low cardiac rehabilitation, with only 14% at baseline, could also influence and skew MACE rates. These factors point to the need to tighten controls in study design and to introduce multi-site recruitment to confirm if the current observations have generalizability beyond this study population. A final point to consider with respect to how tools are used; future designs will need to factor in trialing this tool as a PRO tool in addition to health staff use.

**What is new and important**

1. The SF tool can identify poor self-managers.
2. The SF tool can be used in many health encounters for triaging risk based on self-management capabilities.
3. A binary scoring system can be used in short-form tools.
4. Important gaps exist in identifying higher levels of self-management.

## 5. Conclusions

The **SELFMAN-HF**, for the first time, tested an ***SF*** tool against validated ***LF*** tools in the Flinders Program in HF. This study, importantly, has highlighted the potential to simplify CDSM tools for the targeted purpose of risk stratification and triage in patients with HF. Poor self-mangers, a vulnerable cohort, can be identified with the risk correlating to both readmissions and MACE. Trends for good self-managers are noted. More than half of the cohort who were of borderline and average capabilities could not be risk-stratified against the gold standard. This early finding requires more robust interrogation to assess the short-form tool as a PRO tool, along with its validity, reliability, sensitivity, and specificity in HF, as well as in a spectrum of chronic diseases. CDSM remains a challenging area.

## Figures and Tables

**Table 2:** Baseline descriptive statistics of cohort sociodemographic and clinical variables.

Variable	Cohort (*n* = 117)	%
Age (yo)	66.8 mean	SD 13.5
Sex, (men)	88	75
Ethnicity		
Caucasian	90	77
South Asian	7	6
Asian	3	3
African	10	8
Aboriginal/Pacific Is	7	6
Marital Status		
Married	75	64
Divorced/Separated	23	19
Widowed	15	13
Never married/Single	4	3
Lives/Support		
Alone	46	39
With spouse/Family	71	61
Education Level		
Less than high school graduate	43	36.7
High school graduate	47	40.2
Community college education	18	15.4
Baccalaureate graduate	9	7.7
Graduate school	0	0
Body Mass Index (BMI) (kg/m^2^)		
BMI < 20	6	5
BMI < 25 (Normal)	21	17.9
25 ≤ BMI < 30 (Overweight)	32	27.4
30 ≤ BMI < 35 (Obese 1)	27	23.1
BMI ≥35–40 (Obese II)	14	12
BMI > 40 (Obese III)	17	14.6
Smoking History		
No	53	45
Ex/Yes	64	55
Comorbidities		
CRI		
1 > 60	69	59
30–60	39	33.3
15–30	7	6
<15	2	1.7
CAD	51	44
DM	42	36
HT	79	68
Chol	73	62.4
OSA	31	26.5
Years with HF Diagnosis		
Less than 1 year	83	71
1–4 years	21	18
5–10 years	13	11
LVEF (%)		
Grade 2 (40–49)	1	0.8
Grade 3 (30–39)	84	71.2
Grade 4 (20–29)	29	25
Grade 5 < 20	4	3
Number of Comorbidities		
Causative/nil	30	25.6
>1	26	22.3
>3	59	50.4
>6	2	1.7
* Comorbidity		
IHD	37	31.6
Viral Idio	21	18
AF/Rhythm	31	26.5
Others (VHD/Met Obese, OSA/Chemo-Ca-amyloid)	2/4/4	8.5
A/D	9/9	15.4
BP (mmHg)		
<100/60	42	36
>100/60	75	64
Medications		
Aspirin	69	59
NOAC	47	41
ACEI/ATRA	34	29.1
ARNI	77	66
BB	112	96
SGLT-2	109	93
Statin	74	63
MRA	46	39
Diuretic	67	57
COVID-19 vaccine	98	84
Number of prescribed medications		
≤5	37	32
>5 ≤ 10	57	47
>10	24	21
CR		
Within 6 months of diagnosis	16	14
nil	102	86

* Some have two causes; the first documented aetiology is stated. Abbreviations: ACEI—novel oral anticoagulant; A/D—alcohol and illicit drugs; AF—atrial fibrillation; ATRA—angiotensin receptor antagonist; ARNI—angiotensin receptor neprilysin inhibitor; BB—beta-blocker; BMI—body mass index; BP—blood pressure; Ca—cancer; CAD—coronary artery disease; Chemo—chemotherapy; Chol—hypercholesterolemia; CR—cardiac rehabilitation; CRI—chronic renal impairment; DM—diabetes mellitus; Ex—ex-smoker; HF—heart failure; HT—hypertension; Idio—idiopathic; Is—island; kg/m^2^—weight and height in kilograms and meters; LVEF—left ventricular ejection fraction; Met—metabolic; MRA—mineralocorticoid receptor antagonist; NOAC—novel oral anticoagulant; OSA—obstructive sleep apnea; SGLT-2—sodium–glucose co-transporter inhibitor; yo—years old; VHD—valvular heart disease.

**Table 3:** Summary of health scores at baseline.

Variable	Cohort (n = 117)	Cohort %
NYHA classification at discharge		
I	0	0
II	73	62.4
III	41	35
IV	3	2.6
PHQ-9		
1–4	36	31.5
5–9	27	23.1
>9	52	44.4
SF-12 score, mean (SD)		
Physical Component Summary > 50	4	3.5
Mental Component Summary > 42	69	59
Charlson comorbidity index		
0	0	0
Mild (<2)	27	23.1
Moderate (>2 < 4)	25	21.4
Severe (>5)	65	55.5

See Appendix A, Appendix B, Appendix C for guide to interpreting scores. Abbreviations: n—number of patients; NYHA—New York Heart Association; PHQ—Patient Health Questionnaire; SD—standard deviation; SF-12—Short-Form Survey.

**Table 4:** Comparison of actual and predicted readmission using combined SF scores.

Self-Management Variable *	Self-Management SCORE #	*n* = 117	%	Mean	SD	Range
CFPI						
**#K, M (16/96)**	K					
**0 Good** **≥** **12**	0	6	5.1	6.12	2.41	0–12
**1 Ave** **≥** **9–11**	1	10	8.5			
**2 BL = 6–8**	2	49	41.9			
**3 Poor** **≤** **6**	3	53	45.3			
**#P, C (32/96)**	P					
**0 Good** **≥** **20**	0	33	28.2	17.67	5.27	5–35
**1 Ave** **≥** **17–20**	1	33	28.2			
**2 BL = 12–16**	2	38	32.5			
**3 Poor** **≤** **12**	3	13	11.1			
**#P,C,K,M (96/96)**	M					
**0 Good** **≥** **56**	0	19	16.2	8	2.62	2–14
**1 Ave** **≥** **49–56**	1	19	16.2			
**2 BL = 41–48**	2	64	54.7			
**3 Poor** **≤** **40**	3	15	12.8			
**#KC & PM (48/96)**	C					
**0 Good** **≥** **30**	0	17	14.5	15.75	4.01	5–24
**1 Ave = 24–30**	1	24	20.5			
**2 BL = 18–23**	2	62	53			
**3 Poor** **≤** **18**	3	14	12			
	KC			21.9	5.67	9–36
	0	15	12.8			
	1	27	23.1			
	2	48	41.9			
	3	27	22.2			
	PM					
	0	35	29.9	25.67	7.58	7–45
	1	43	36.8			
	2	26	22.2			
	3	13	11.1			
	Total					
	***0***	*30*	*25.6*	47.55	12.7	16–79
	1	21	17.9			
	2	31	26.5			
	*3*	*35*	***29.9***			
* SF						
0 Good **	***0***	***13***	***11.1***	1.71	1.04	0–3
**1 Ave**	1	46	39.3			
**2 BL**	2	20	17.1			
**3 Poor**	***3***	***38***	***32.5***			
**1. Ma (K+C)**	***0***	76	65	na	na	na
**2. Mx (P+M)**	***0***	61	52.1	na	na	na
3. Mse (K+C+P+M) **	***0***	***13***	***11.1***	na	na	na

This table highlights the combined ***SF*** scores for patients compared to the combined ***LF*** scores for the matching self-management domains, as well as the total ***LF*** score. ** All patients who had good self-tailoring were good in terms of the ***SF* score**. No patient who scored poorly in ***LF*** scored well in ***SF***, although the ***SF*** under-identified good patients, with 13 (11.1%) identified in ***SF*** and 30 (25.6%) identified in ***LF***. Poor self-managers aligned closer with 38 (32.5%) versus 35 (29.9%). * Self-management variables: (a) ***LF*** has four domains: C—coping; K—knowledge; M—recognition and management of symptoms; P—partnership in treatment; (b) ***SF*** has three domains: Ma—self-maintenance/monitoring; Mx—self-management/tailoring; Mse—self-efficacy or good chronic disease self-management; (c) Combining scores: domains are combined to match and compare the ***SF*** and ***LF***. Domains that overlap are (1) Ma = K + C; (2) Mx = P + M; (3) Mse = K + C + P + M. # Self-management scores (first row): (a) ***LF*** questions are scored from 0 to 8. Two domains (K,M) have two questions and combined scores range from 0 to 16; two domains have four questions and combined scores range from 0 to 32; the total score is 96. Different combinations for ***LF*** scores are compared to the ***SF*** score to match self-management capabilities. (b) The ***SF*** domain is scored 0 or 1, and the total score varies from 0 to 3. Abbreviations: Ave—average and above; BL—borderline; ***LF—***Flinders Program of chronic condition management (Flinders Program or FP); na—not applicable; SF—Screen in Heart Failure Tool (SCRinHF).

**Table 5:** SF and LF correlation with readmission and MACE.

Self-Management Score		Events				
SF Score/(n)	LF (n) Mean Range	Clinic Appointments/ FTA (>25%)	CV Adm HF Adm	NCV/Multiple	S/E	Deceased (*n* = 6)
0 (13)	30	21.3 (10–34)	5 *	5/13	2	Nil
	59.8					
	24–76	3	4			
3 (38)	35			29	24	
	36.13	22.7 (4–48)	31 *			4
	16–53	17	25			32.4 (16–47)

Poor self-managers identified by ***SF*** and ***LF*** had significantly more cardiovascular admissions than good self-managers. * Statistically significant association with the χ^2^ (chi squared = 8.69) test at 1 degree of freedom between the observed ***SF*** score of 0 (mean ***LF*** 59.8) and heart failure admissions compared to the ***SF*** score of 3 (mean ***LF*** 36.13) *p* < 0.01. Abbreviations: Adm—admission; ***LF***—Flinders Program of chronic condition management; CV—cardiovascular; FTA—failure to attend clinical review (>25% of appointments); n—patient numbers NCV—non cardiovascular; ***SF***—Screen in Heart Failure tool (SCRinHF); S/E—side effect. NB// Figure 2 is graphical representation of Table 5 results.

## Data Availability

We abide by the data sharing policy. Details of CFPI and SCRinHF can be obtained from https://www.frontiersin.org/articles/10.3389/fmed.2023.1059735/full.

## Appendix B. Heart Failure and Illness Burden of Cohort (Table 1 and Table 2)

To interpret the table, the description of the cohort is provided. At baseline, all patients had commenced SGLT-2i within 6 months of identification for study enrolment. At this point, at least three of the four HF pillars were already started in patients diagnosed prior to 12 months. Most patients with a diagnosis of HF within the 12-month period were also optimized on CHF pillar therapy [beta-blocker, renin–angiotensin aldosterone, and/or neprilysin inhibitor (RAAS/ARNI) or mineralocorticoid receptor antagonist (MRA)]. As was the requirement for eligibility of SGLT-2i, all patients were NYHA class II or more, with left ventricular ejection fraction (LVEF) class III (<40%) and greater. HF therapy was good; all patients were on antiplatelet or anticoagulant at baseline. HF pillars were 112 (96%) for beta blockers, 111 (95.1%) for RAAS/ARNI, and 46 (39%) for MRA. Polypharmacy was common: 101 (68%) had at least five prescribed medications classes. Cardiac rehabilitation was only recorded among 16 (14%) patients. At baseline, patient symptoms were NYHA class II in 73 (62.4%) and 41 (35%) NYHA class III. Only three patients were NYHA IV from the urgent referrals, as is expected for community outpatients. On imaging, 1 patient (0.8%) had grade 2 LVEF, while 84 (71.2%), 29 (25%), and 4 (3%) had grades 3, 4, and 5, respectively. The etiology of HF was most commonly ischemic heart disease (IHD) (37, 31.6%), followed by atrial fibrillation (AF) (31, 26.5%) and viral or idiopathic diseases (21, 18%). Patients with drug (amphetamine) (9) and alcohol abuse (9) accounted for 18 cases (15.4%); miscellaneous combination of metabolic syndrome, valvular heart disease, amyloid, and chemotherapy accounted for several cases individually, totaling 10 patients (8.55%). Blood pressure was lower than 100/60 mmHg in 42 (36%) patients, although this study was not equipped to report on the impact of drug dosing and delivering guideline-based medical therapies (GDMT). A limited number (16m 14%)—predominately IHD—who received treatment underwent cardiac rehabilitation within 6 months of their recorded diagnosis. Health scores for depression with PHQ-9 were negligible for 36 patients (31.5%), intermediate for 27 (23.1), and high risk for 52 (44.4%). In comparison, the SF-12 highlighted 48 patients (41%) at high risk. The SF-12 physical score highlighted high physical disability in the cohort for 113 (96.5) patients, with moderate or greater limitations. The Charlson Comorbidity Index (CCI) highlighted an elevated comorbidity risk, with mild, moderate, and severe scores for 27 (23.1%), 25 (21.4%), and 65 (55.5%) patients, respectively, with the older cohort having higher scores.

## Appendix C. PHQ-9 Scores

Reference: Kroenke et al., 2001 [29].

## Appendix D. SF-12 Scoring

Scores range from 0 to 100, with higher scores indicating better physical and mental health functioning [28]. A score of 50 or less on the PCS-12 has been recommended as a cut-off to determine a physical condition, while a score of 42 or less on the MCS-12 may be indicative of “clinical depression”

Reference: Soh et al., 2021 [30].

## Appendix E. Charlson Comorbidity Score

A cut-off of 50 or less can be used to identify a physical condition, while a score of 42 or less may signify clinical depression.

Reference: Charlson et al., 1987 [31].
